# Stand-Biased Desk Intervention on Sleep Quality of High School Students: A Pilot Study Using Tri-Axial Accelerometery

**DOI:** 10.3390/ijerph17010037

**Published:** 2019-12-19

**Authors:** Joohyun Rhee, Mark E. Benden

**Affiliations:** Department of Environmental and Occupational Health, School of Public Health, Texas A&M University, College Station, TX 77843-1266, USA; mthowdy@tamu.edu

**Keywords:** physical activity, standing desk intervention, sleep pattern

## Abstract

Prolonged sitting is related to a sedentary inactive lifestyle and related to obesity and many metabolic problems caused by inactivity. The problem gets more serious for people who spent most of their work time in a seated position like students or office workers. In this study, we provided standing desk and stool to the local public high school and observed the changes in their behavior in terms of physical activity using tri-axial accelerometer before and after intervention. Previously published study using the same dataset under the larger project reported increased physical activity during school hours. In this study, we extracted more diverse features directly from the raw data instead of using data processed by the software that manufacturer provided. Hence, we were able to analyze the same features (sedentary, physically active time) as well as sleep-related variables. Of the interest, sleep is another important feature that can tell us about participants’ health conditions. Even if the intervention contributed to updating their behavioral patterns, the result might be nullified in the long run if their sleep pattern was compromised. The quantity and quality of sleep was not changed after the intervention. Therefore, the efficacy of standing desks has been confirmed again.

## 1. Introduction

As the prevalence of obesity has increased rapidly worldwide [1], a sedentary lifestyle, one of the major causes of obesity and overweight, also has increased [2]. The amount of sedentary behavior during waking hours is especially high for office workers and students [3]. Obesity is a cause of numerous health problems such as a declined mobility [4], increased fatigability [5], and decreased brain volume [6]. Although the aforementioned studies investigated the effect of obesity on older populations, being obese early in life is known to affect adult obesity as a result of accumulated damage to the body [7]. Efforts to reduce sedentary behavior and its resulting health burden is an emerging field of research.

One feasible intervention to improve excessive sedentary behavior is to use a stand-biased desk. Despite the fact that increasing physical activity with exercise would be our first choice to overcome any inactivity related health issues, using a standing desk is an intervention strategy that an inactive population can accept with less difficulty due to its feasibility [8]. Stand-biased desks also act as a proxy for movement/ambulation as demonstrated in recent studies [9]. Studies also reported that standing desks improved health in the workplace [10] and in the classroom [11,12].

In the present study, we observed the physical activity of high school students before and after a stand-biased desk intervention using a 3-axis accelerometer (Activpal, Pal Technologies, Glasgow, UK). We analyzed the same data set used in the previous study that demonstrated an improved physical activity during school hours as a result of the conversion to stand-biased desks [9]. However, we expanded the scope of analysis from the physical activity during the 8 h window of school hours to 24 h window that includes sleep period. The present study is the first to also examine the effect of a stand-biased desk intervention on the quantity and quality of sleep. In addition to physical activity, sleep is another element that affects a person’s overall health. Moreover, sleep is not only a rest period to recover from fatigue accumulated during the wake period, it affects a person’s memory and learning. It is well known that sleep duration is related to health [13] and obesity prevalence in children and adults [14].

The purpose of the present study was to evaluate the effect of using stand-biased desks in the classroom during school hours on physical activity of entire day and sleep quality and quantity of high school students. We hypothesized that the stand-biased desk intervention reduced sedentary behavior during awake hours and improved the quality of sleep.

## 2. Materials and Methods

### 2.1. Participants

A total of 23 first-year high school students were recruited from a local public high school. The student’s entire school was converted at the winter break from traditional seated desks and chairs to stand-biased desks with stools. The participants were randomly selected from a total of one hundred high school students recruited for a larger study on sedentary behavior. Participants were paid USD 25 upon completion of two data collection sessions, one in the fall semester before the standing desk intervention and another one in the spring semester after the intervention. All participants provided assent and written parental consent prior to their participation. The study protocol was approved by Texas A&M University’s Institutional Review Board (IRB 2015-0096D) and the data collection, and the intervention was approved by the Bryan Independent School District.

### 2.2. Procedures

Participants’ physical activity was monitored using the 3-axial accelerometer for 3 consecutive 24 h days (ActivPal, Pal Technologies, Glasgow, UK). The Activpal physical activity monitor was attached to each participant’s right thigh using a Tegaderm film (3M Company, Maplewood, MN, USA) after being sealed for water resistance using a finger cot wrapped with another Tegaderm film. The physical activity data were collected two times before and after the standing desk intervention. None of the participants had previous exposure to stand-biased school environments. This group of students used traditional seated desks and chairs in the classroom during school hours for the period of the first data collection in the fall. The second data collection occurred in the following spring semester after participants had been using stand-biased desks in the classroom for approximately three months.

### 2.3. Data Process and Analysis

Raw 3-axis acceleration signals recorded with 20 Hz sampling frequency were extracted from the Activpal physical activity monitors. The jerk, a time derivative of acceleration, of each axis was computed and the absolute sum of each 15 s window was acquired. A time window that did not show a change of acceleration for longer than 5 min in any axis was automatically marked as sleep [15]. Bedtime and wake time marked by a computer algorithm detecting a period of inactivity automatically was later confirmed by visual inspection of acceleration data. Acceleration data of wake and sleep periods were processed separately. Total time spent on each physical activity level of sedentary, stand, and step was obtained from the data of wake period. The number of body movements was obtained from the data of sleep period. The body movements on the transverse plane of a participant, which are a change between supine and prone position, or between lateral position and the other two were defined as body turns. The movements on the sagittal plane of a participant was defined as leg movement since the sensor was attached on the participant’s right thigh parallel to the coronal plane. Acquired dependent variables were submitted to the mixed factor analysis of variance to evaluate sex-related effects of the intervention. Processing of acceleration data to acquire the dependent variable was done using Matlab 2018b (Mathworks, Natick, MA, USA), and statistical analysis was performed using IBM SPSS version 25 (IBM corporation, Armonk, NY, USA).

## 3. Results

Of the 23 recruited participants, 19 completed two data collection sessions (one pre and one post-intervention) of at least 72 consecutive hours each, seven males and 12 females; mean height 164.4 cm (SD 8.4), mean weight 60.3 kg (10.6), and mean body mass index 22.3 kg/m^2^ (3.8). Table 1 provides mean (SD) of height, weight, and BMI data of the participants split by gender and pre/post intervention. Participants’ physique before and after the intervention for each gender was not significantly different. Table 2 shows the average of each data collection session for the wake/sleep duration, physical activity during the wake period, and the number of movements during sleep.

**Figure 1:**
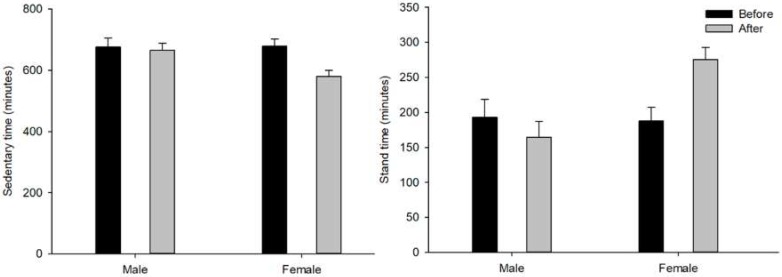
Sedentary time and stand time during the awake period in minutes. The left panel shows the average sedentary time, and the right panel shows the average stand time during awake period. Error bars represent standard error.

Average sedentary time during the awake period was significantly decreased after the intervention (intervention main effect, F (1, 16) = 8.219, *p* = 0.011) and females exhibited greater decrease (intervention x sex interaction effect, F (1, 16) = 5.234, *p* = 0.036; Figure 1). The average stand time during wake period was increased after intervention but was not statistically significant (Intervention main effect, F (1, 17) = 3.147, *p* = 0.094). Females showed significant increased standing time while males showed no difference (intervention x sex interaction effect, F (1, 17) = 12.281, *p* = 0.003). Females exhibited marginally significant longer stand time (sex main effect, F (1, 17) = 4.345, *p* = 0.053; Figure 1). Step time during wake period was not different (all *p* > 0.2).

**Figure 2:**
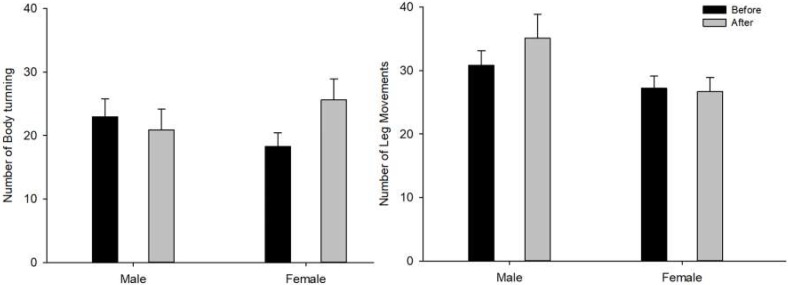
Body movement during sleep before and after the intervention. The left panel shows the number of body turns, and the right panel shows the number of leg movements. Error bars represent standard error.

Average sleep or awake period in minutes after the stand-biased desk intervention was not changed (all *p* > 0.5). The number of body turns during the sleep period was not changed after the intervention and no sex-related difference was observed (Figure 2). However, marginally significant intervention x sex interaction (F (1, 17) = 3.541, *p* = 0.077) indicates a possible sex-dependent difference in change patterns. Simple main effect analysis revealed that the number of body turns during sleep was increased after the intervention for females while males showed slightly decreased numbers of body turns that were not significant. The number of leg movements during the sleep period was not changed with the intervention but leg movements for males were greater than females (sex main effect, F (1, 17) = 4.499, *p* = 0.049; Figure 2). 

## 4. Discussion

In the present study, we examined the effect of a stand-biased desk intervention on high school students’ behavior and sleep patterns. Similar to the result of the previous study that analyzed the same data set for behavior pattern changes during school hours [9], the present study found significantly decreased sedentary behavior time and somewhat increased stand time during the wake period. Interestingly, females exhibited greater changes in sit and stand time than males with the stand-biased desk intervention during school hours. Analysis of sleep-related variables showed marginally significant group dependent changes. Although significant changes in mean weight after the intervention were observed, body mass index was not changed; thus, weight increases seem connected to normal growth.

The decreased sedentary behavior with a stand-biased desk intervention is congruent with other studies that reported decreased sedentary behavior and reduced energy expenditure [12,16,17]. Moreover, the female group showed greater improvement in behavior time, but it seems inconclusive as this sex-related change in behavior may not be solely caused by the intervention. A stand-biased desk can be an effective and scalable method to improve inactive lifestyles among students. However, research shows that prolonged standing might also increase the stress on the lower back and joints, especially to over-weight or obese people [18,19]. Therefore, development of a strategy to properly distribute the time to sit, stand, and walk is recommended to promote the best use of these interventions and to improve the health of inactive people. More recent research has shown that the use of prompting via computers may help us remember to make postural changes throughout the day in workstations equipped with stand-capable desk [20].

The decreased sedentary behavior caused by a stand-biased desk intervention may accompany neurocognitive benefits. The present pilot study was a part of the larger project that tested the effect of stand-biased desk intervention in high school with up to 100 students. Mehta et al. [11] reported the improved cognitive executive functions of high school students after the stand-biased desk intervention in their pilot study that was also a part of the same larger project. Although the two studies did not share the participants, assuming the effect of the stand-biased desk was similar across participants in the project, it is probable that the improved behavioral outcome that current study reports and the improved cognitive function that Mehta et al. [11] reported are related. However, a further study that evaluates behavioral and cognitive outcome changes related to the stand-biased desk intervention is warranted.

Contrary to our hypothesis, the stand-biased desk intervention during school hours did not affect the quantity and quality of sleep. The result suggests that the effect of increased physical activity due to the stand-biased desk intervention on sleep was not positive that aerobic exercise would provide [21] nor negative indicating a compromised sleep pattern caused by increased fatigue [22]. Short sleep duration causes cognitive function declines during the wake period and increased obesity prevalence in children and adults [13,14,23]. Despite the significant result observed in physical activity, the participants in the current study slept 8 hours on average, and the sleep duration was not changed after intervention. Two types of body movements during sleep, the number of body turns and leg movements, were extracted from the raw acceleration data and analyzed to assess the quality of sleep. The number of body turns was not changed after the intervention. For the number of leg movements, males showed greater numbers of leg movements than females. Frequent body movements are known to be a sign of sleep-related problems [24]. For example, leg movements greater than 50~60 times a night is normally considered to be restless leg syndrome [25]. The number of body movements in the current study was in the normal range, and the result that showed no significant changes with the stand-biased desk intervention may indicate the intervention had no effect on the quantity and quality of the sleep. This dispels a significant concern raised by researchers that compensatory behaviors after school or during sleep could erase the positive effects of extra movement during the school day that the intervention caused.

A number of limitations exist in the study. First, a small sample size (*n* = 19) could not provide enough statistical power to test all the effects. Second, no control group was present in this study. It was due to the fact that we assumed our participants who were non-athletic high school students share a similar lifestyle, but there is a possibility of weakening the certainty of the stand-biased desk intervention effect reported in the present study. Third, extracted dependent variables from raw acceleration data provide reasonable measures but cross-validation against other validated measures will make the observed data more compelling and generalizable. Fourth, the results of this study assume any measured effect to be that of the intervention. However, the results and conclusion could also have been impacted by the cofactors reflecting the participants’ lifestyle such as physical activity pattern, diet, emotional health status, sleep hygiene, behavior before the bedtime, cognitive and physical performance before and after the intervention. Future studies should examine the efficacy of the stand-biased desk using more common sleep study settings and controls.

## 5. Conclusions

In conclusion, the result of this pilot study provides evidence of positive effects of standing desk intervention. While the participants showed increased physical activity after intervention, their quality and quantity of sleep were not changed. This result suggests a standing desk is a feasible intervention method that contributes improving sedentary inactive lifestyle. 

## Figures and Tables

**Table 1:** Participant demographics (Mean (SD)).

Demographics	Female (*N* = 12)			Male (*N* = 7)		
	Before	After	*p*	Before	After	*p*
Height (cm)	161.5 (7.2)	162.7 (6.4)	0.658	169.5 (8.3)	171.1 (8.7)	0.727
Weight (Kg)	63.5 (11.0)	64.8 (10.9)	0.779	57.3 (7.9)	57.3 (8.6)	0.577
BMI (kg/m^2^)	24.3 (3.3)	24.4 (3.4)	0.925	19.0 (1.9)	19.5 (2.2)	0.642

**Table 2:** Summary of dependent variables. The average duration of sleep, wake, sedentary, stand and step in minutes, and the number of body turns and leg movements during the sleep period.

Descriptive	Sleep		Wake		Sedentary		Stand		Step		Turning		Leg Movement	
	Before	After	Before	After	Before	After	Before	After	Before	After	Before	After	Before	After
**Mean**	483.66	487.58	956.33	952.41	680.75	617.28	187.35	228.56	77.49	72.62	19.02	25.51	29.09	30.06
**Std. Dev**	45.73	54.64	45.73	54.64	70.28	73.74	62.20	82.65	23.31	26.78	7.01	12.26	5.91	8.79
**Minimum**	396.56	367.63	880.45	870.80	554.73	476.60	75.57	58.14	45.34	13.26	9.33	10.66	17.33	16.33
**Maximum**	559.55	569.20	1043.43	1072.36	789.01	736.89	328.24	394.52	119.77	121.22	34.33	57.00	39.00	44.00
